# Three-Dimensional Printed Antimicrobial Objects of Polylactic Acid (PLA)-Silver Nanoparticle Nanocomposite Filaments Produced by an In-Situ Reduction Reactive Melt Mixing Process

**DOI:** 10.3390/biomimetics5030042

**Published:** 2020-09-02

**Authors:** Nectarios Vidakis, Markos Petousis, Emmanouel Velidakis, Marco Liebscher, Lazaros Tzounis

**Affiliations:** 1 Mechanical Engineering Department, Hellenic Mediterranean University, Estavromenos, 71004 Heraklion, Crete, Greece; vidakis@hmu.gr (N.V.); m.velidakis@gmail.com (E.V.); 2 Institute of Construction Materials, Technische Universität Dresden, DE-01062 Dresden, Germany; marco.liebscher@tu-dresden.de; 3 Department of Materials Science and Engineering, University of Ioannina, 45110 Ioannina, Greece; latzounis@uoi.gr

**Keywords:** Fused Filament Fabrication (FFF), 3D printing, reactive melt processing, antimicrobial (AM) properties, polylactic acid (PLA), silver (Ag) nanoparticles (NPs), polyethylene glycol (PEG), polyvinyl pyrrolidone (PVP), *Staphylococcus aureus* (*S. aureus*), *Escherichia coli* (*E. coli*)

## Abstract

In this study, an industrially scalable method is reported for the fabrication of polylactic acid (PLA)/silver nanoparticle (AgNP) nanocomposite filaments by an in-situ reduction reactive melt mixing method. The PLA/AgNP nanocomposite filaments have been produced initially reducing silver ions (Ag^+^) arising from silver nitrate (AgNO_3_) precursor mixed in the polymer melt to elemental silver (Ag^0^) nanoparticles, utilizing polyethylene glycol (PEG) or polyvinyl pyrrolidone (PVP), respectively, as macromolecular blend compound reducing agents. PEG and PVP were added at various concentrations, to the PLA matrix. The PLA/AgNP filaments have been used to manufacture 3D printed antimicrobial (AM) parts by Fused Filament Fabrication (FFF). The 3D printed PLA/AgNP parts exhibited significant AM properties examined by the reduction in Staphylococcus aureus (*S. aureus*) and Escherichia coli (*E. coli*) bacteria viability (%) experiments at 30, 60, and 120 min duration of contact (*p* < 0.05; *p*-value (*p*): probability). It could be envisaged that the 3D printed parts manufactured and tested herein mimic nature’s mechanism against bacteria and in terms of antimicrobial properties, contact angle for their anti-adhesive behavior and mechanical properties could create new avenues for the next generation of low-cost and on-demand additive manufacturing produced personal protective equipment (PPE) as well as healthcare and nosocomial antimicrobial equipment.

## 1. Introduction

Poly-Lactic Acid (PLA) which belongs to the family of polyglycolic acid aliphatic polyesters has received an extensive interest the last decades especially as a high-performance polymer for bio-related applications. More specific, PLA has been used in the biomedical sector for various functional objects i.e., implants [1], surgical equipment [2], nanofibrous templates for drug delivery [3], foams for tissue engineering [4], etc. PLA is a thermoplastic in nature polymeric material known also for its biocompatibility, biodegradability, superior mechanical strength compared to other thermoplastics, and ease of processing via solution and melt processing methods [5]. Moreover, for biomedical applications as for instance protective personal equipment (PPE), surgical equipment, etc., PLA offers the unique property that it can be sterilized due to its relatively high melting point (typical T_m_ of PLA ~150–160 °C), which is a prerequisite so that PLA based 3D objects can be reusable till the end of their lifetime.

3D printing is a technology in which parts are produced with the sequential addition of material layers. It belongs to the family of the additive manufacturing technologies and has received extensive scientific interest over the years. Additionally, 3D printing is in the forefront amongst other additive manufacturing technologies for on demand product development. In addition to that, 3D printing offers the unique possibility of producing 3D bulk objects consisting of different materials with various physical and chemical properties [6,7]. For these and several other practical reasons, 3D printing and additive manufacturing in general have been adopted with fervor by industry and are increasingly used as production processes. Various techniques are available nowadays for 3D printing solid materials, including Fused Filament Fabrication (FFF), direct metal laser sintering and electron beam fabrication [8]. 3D printing has been employed recently to manufacture surgical equipment [8,9], implants [10], tissue scaffolds [11], 3D bioelectronics [12], etc.

One of the most promising applications of 3D printing and especially FFF technology is amongst others in the medical field for the design and development of medical devices and instruments [13,14]. Moreover, surgeons have used patient-specific computed tomography derived 3D prints for the better preoperative planning and the proper design of the surgical approach in complex operations [15,16,17]. In the same philosophy, 3D printed models have been used also for educational proposes of young surgeons [18,19]. Although the mechanical properties of the 3D printed have been extensively studied in literature [20], there is, however, scant literature to date for the production of multi-functional 3D printed objects, i.e., with enhanced mechanical properties [20] and additionally electrical conductivity [21], thermoelectric property [22], anti-adhesive and antimicrobial properties to avoid biofilm formation [23], etc., utilizing novel and functional materials.

Recently, a great scientific focus has been devoted to the development of bulk materials with antimicrobial (AM) properties, specifically for various applications in the health sector so as to increase the environment’s hygiene and avoid the transmission and infections caused by pathogenic microorganisms [24]. Polymers as a big family of plastic materials of which many bulk 3D objects are consisting of in applications such as hospital tables, protective equipment, paints, biomedical products like implants, bandages, catheters, surgery equipment, etc. have been modified by antimicrobial agents in their bulk structure via melt-mixing or solvent mixing methods. Moreover, another promising approach to endow AM properties to bulk 3D plastic components is by depositing AM films onto their outer surface by: (i) either wet chemical methods (dip-coating using AgNP dispersions [25], sonochemical immobilization [26,27], etc.) or (ii) vacuum deposition techniques (magnetron sputtering [28], ion-beam-assisted deposition process [29], etc.). To that end, a great number of AM agents as for instance copper nanoparticles (CuNPs) and/or copper complexes, silver NPs (AgNPs) and Ag metal salts, polyhexamethylene biguanides, triclosan and chitosan based biopolymers, quaternary ammonium compounds, etc. have been reported and proven to prevent the growth of pathogenic microorganisms, such as bacteria, fungi, algae, etc. [30,31,32] upon being incorporated/blended into the polymer 3D bulk structure or deposited as thin films [33,34].

Amongst others, AgNPs have been in the forefront of research and several times reported for their antibacterial activity, especially due to their broad spectrum of antibacterial activity while at the same time having proven low levels of toxicity against mammalian cells [35]. However, their antimicrobial activity has not yet been fully understood. Relatively, one of the most plausible mechanisms is initially the interaction of AgNPs with the microorganism’s surface that results further to the penetration of AgNPs and/or Ag released ions (Ag^+^) through the cell walls, allowing them to react with the thiol group of proteins and ending up with the cell’s distortion and death [36]. Silver in its ionized form is highly reactive, as it binds to tissue proteins and endows structural changes to the bacterial cell wall and nuclear membrane, leading thus to cell lysis and death [37]. A side reaction of AgNPs concomitantly to their interaction with bacteria cells and bactericidal mechanism, is unavoidably the direct interaction with human cells, which leads to cytotoxicity and genotoxicity reported effects [38]. As such, it is of crucial importance AgNPs to be stabilized and/or embedded in the bulk structure of 3D component materials. Therefore, polymer/AgNP nanocomposites can be promising antimicrobial materials, which can be easily processed, i.e., via melt mixing and extrusion processes. Furthermore, polymer/AgNP nanocomposites can be extruded in the form of filaments that can be utilized further to 3D print complex and on-demand antimicrobial 3D printed bulk parts through FFF additive manufacturing technology. In relation to that, the incorporation of AgNPs in a polymer matrix by solvent mixing has been already reported [39].

The production of polymer nanocomposites by well-established industrial and large-scale processing methods as for instance melt compounding using an extruder is very advantageous [40,41,42,43]. A very promising approach to fabricate polymer/AgNP nanocomposites is via reactive melt mixing, since preformed AgNPs found as powders tend to agglomerate. The aggregation phenomena are known further to affect the material’s properties, i.e., their antimicrobial efficacy in since the interaction between the bacterial cell and the AgNPs is more intensive if the silver nanoparticles are well dispersed and not agglomerated [44]. In that method, the silver precursor is solubilized in the polymer melt during processing, and either the selected polymer matrix exhibits the functional groups to reduce in-situ the silver salt into elemental silver or some other polymeric material is blended and utilized as the reducing agent. For instance, it has been already reported the generation of AgNPs in a thermoplastic polyurethane matrix (TPU) by in-situ reduction of silver acetate during polymer melt and extrusion processing, while the developed materials have exhibited antimicrobial properties [44]. AgNPs have been formed also via in-situ reduction during melt mixing in a poly(methyl methacrylate) PMMA matrix as well as in different polyamides [45,46]. Finally, Parida et al. [47] recently reported on a solventless in-situ reduction method during the extrusion process to prepare AgNP using different silver precursors and thermoplastic polymers for antimicrobial food packaging application. Moreover, they used the same protocol to synthesize AgNPs in polylactic acid (PLA) and polypropylene (PP), and they found that the surface energy of polymer melt had an effect on the quality and size of AgNPs created.

It has been previously reported that the surface topography and roughness may have a great influence on the bacteria attachment and further colonization via the formation of a self-produced polysaccharide based biofilm [48]. The factors that dictate the underlying mechanism of “attachment” include: (i) surface hydrophobicity, (ii) electrostatic interactions, (iii) van der Waals forces, and (iv) steric hindrance [49]. In specific, the hydrophobicity is a surface property that can be engineered via mimicking the micro- and nanostructure of naturally occurring surfaces such as cicada and dragonfly wings, lotus leaves, and shark skin [48]. Moreover, the nano and micro-scale hierarchical structure on lotus leaves are responsible for its unique superhydrophobic and self-cleaning properties [50]. 3D FFF printing offers the unique opportunity to create microstructured surfaces, i.e., down to 50 µm printed object layer thickness, while incorporated nanoparticles in the case of using nanocomposite thermoplastic filaments that appear onto the surface of the 3D printed object could result in hierarchical nano-/and micro-scale structures like the structure of lotus leaves.

Herein, the main purpose of this research article is to fabricate high quality PLA/AgNP nanocomposite 3D printing filaments via reactive melt mixing which is a facile, scalable and industrially viable method, while the filaments have been utilized further to manufacture 3D printed samples of different printed layer thickness that have been tested in terms of hydrophillicity, mechanical and antibacterial performance. Specifically, PLA/AgNP antimicrobial filaments have been produced by reactive melt mixing blending: (i) PLA, AgNO_3_ and PEG, as well as (ii) PLA, AgNO_3_ and PVP, while PEG and PVP have been utilized as the reducing agent polymeric material, the PLA as the polymer matric and AgNO_3_ as the AgNP salt precursor. AgNPs have been generated in situ in the PLA polymer matrix, yielding extremely efficient antimicrobial (AM) objects as determined by the reduction in *Staphylococcus aureus* (*S. aureus*) and *Escherichia coli* (*E. coli*) bacteria viability (%) after being exposed for 30, 60, and 120 min, respectively. Different characterization techniques have been carried out i.e., Raman spectroscopy and thermogravimetric analysis (TGA) for the PLA/AgNP nanocomposite extruded filaments, while water contact angle of a sessile drop, optical and scanning electron microscopy (SEM), antibacterial tests, and tensile and micro-hardness tests of the 3D printed respective samples.

## 2. Materials and Methods

**Figure 1:**
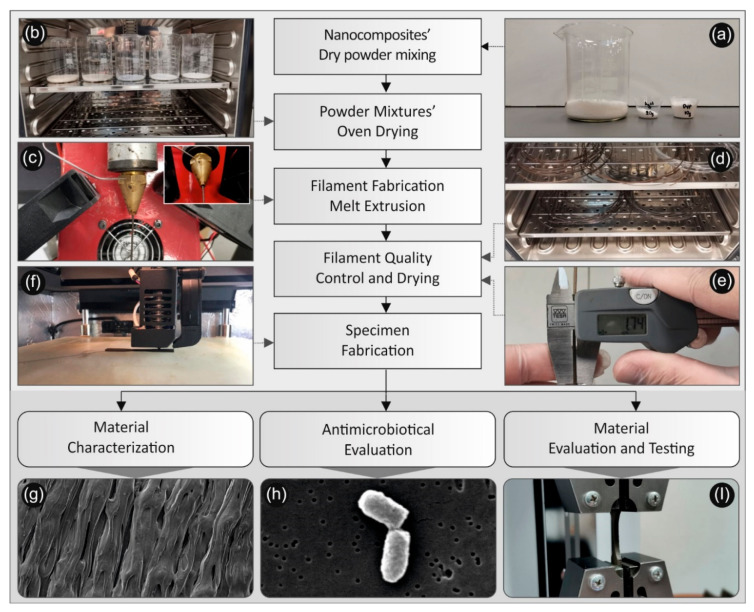
Schematic illustration for the process followed for the creation of poly-Lactic Acid (PLA)/silver nanoparticle (AgNP) nanocomposite filaments, for their characterization as well as the study of their antimicrobial and mechanical properties: (**a**) PLA, AgNO_3_ and PEG or PVP (reducing agents) powders used for the reactive melt mixing, (**b**) the drying process of the powders/ materials before the reactive melt mixing process, (**c**) the PLA/AgNP filament fabrication via melt extrusion process, (**d**) the drying process of the produced PLA/AgNP nanocomposite filaments, (**e**) measurement of the produced filament diameter to be used for the FFF 3D printing process, (**f**) 3D printing FFF process for the manufacturing of the samples, (**g**) SEM image of the 3D printed samples’ side surface showing the additively 3D printed layers of the samples, (**h**) SEM typical image of bacteria attached onto the samples surface after different durations in contact with the antibacterial 3D printed samples used in the antibacterial tests carried out in this work, (**i**) 3D printed dog bone-shaped sample during tensile testing.

The methodology followed in this work for the development of the nanocomposites, their characterization, the study of their antimicrobial and their mechanical properties is shown schematically in Figure 1 and described in detail further below.

### 2.1. Materials

The polymer matrix used in this work was industrial grade PLA (PLA 3052D) fine powder received from Plastika Kritis S.A (Heraklion, Crete, Greece). This specific PLA grade has a 3.3 relative viscosity, 200 °C melting temperature, 55–60 °C glass transition temperature, and a density of 1240 kg/m^3^. Silver nitrate (AgNO_3_, ≥99%), Polyethylene glycol (PEG) with average molecular weight M_n_ ≈ 4600 g/mol and Polyvinyl Pyrrolidone (PVP) with average molecular weight M_n_ ≈ 10,000 g/mol were supplied by Sigma Aldrich (Steinheim, Germany). All the chemical reagents in this study were used as received without further purification.

In this work, PEG has been used as a melt blended additive material co-compounded with the PLA in order to function as a reducing agent of Ag ions (Ag^+^) stemming from mixed AgNO_3_. In specific, the reduction of Ag^+^ by PEG has been proposed to occur through the oxidation of the PEG hydroxyl (-OH) terminal groups to aldehyde groups and the concomitant creation of Ag^0^ NPs, as it has been previously reported in literature [51,52]. On the other hand, PVP that has been utilized also in this work as a melt blended reducing agent material has been reported to function as a reducing agent for the creation of AgNPs via different Ag precursor compounds as well as suitable colloidal stabilizer, surfactant, shape-directing agent and dispersant [53]. The possible interactions of PEG and PVP polymeric additives with the Ag^+^ and further the reduction process occurring by the melt mixing operational temperature (T = 230 °C), which resulted into “in-situ” creation and incorporation of AgNPs within the PLA matrix could be seen in the graphical abstract figure of this work.

### 2.2. Fabrication of PLA/AgNP Nanocomposites by Reactive Melt-Mixing Extrusion Process and 3D Printing of PLA/AgNP Nanocomposite Filaments

Initially, the PLA matrix material was mixed with AgNO_3_ and PVP (Figure 1a), as well as AgNO_3_ and PEG powders, respectively, utilizing a mechanical homogenizer (the Silverson L5M-A laboratory mixer was used as a homogenizer for about 10 min for each batch of the polymers produced.). The powder mixtures were dried then in a vacuum oven (Figure 1b), at 70 °C for 48 h. Specifically, the following mixtures were prepared prior to the reactive melt mixing/compounding extrusion process for the reduction of Ag^+^ to metallic Ag^0^ NPs formed in-situ within the PLA matrix during melt mixing:

1. Recipe-01: 100 g (PLA): 20 g (AgNO_3_): 10 g (PEG), hereafter denoted as PLA/Ag/PEG (rec-01);
2. Recipe-02: 100 g (PLA): 10 g (AgNO_3_): 5 g (PEG), hereafter denoted as PLA/Ag/PEG (rec-02);
3. Recipe-03: 100 g (PLA): 20 g (AgNO_3_): 10 g (PVP), hereafter denoted as PLA/Ag/PVP (rec-03); and
4. Recipe-04: 100 g (PLA): 10 g (AgNO_3_): 5 g (PVP), hereafter denoted as PLA/Ag/PVP (rec-04).

The selection of AgNO_3_ and PEG/PVP respectively masses has been done based on a protocol of Nam et al. [54], without “in-detail” calculation in this work of the molar ratio of OH^−^ groups (PEG) or Pyrrolidinone groups (PVP) to the Ag^+^, since it is not the main aim/focus of this research study.

**Figure 2:**
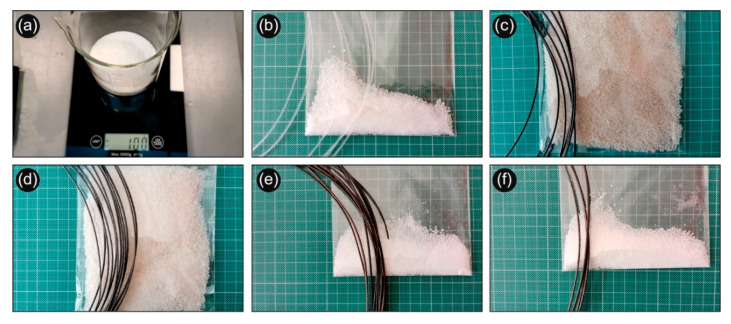
(**a**) Determining the wt. concentration for the creation of the different recipes (**b**) Neat PLA powder and the filament produced with this powder (**c**) 100 g (PLA): 20 g (AgNO_3_): 10 g (PEG), hereafter denoted as PLA/Ag/PEG (rec-01) powder and the filament produced with this powder (**d**) 100 g (PLA): 10 g (AgNO_3_): 5 g (PEG), hereafter denoted as PLA/Ag/PEG (rec-02) powder and the filament produced with this powder, (**e**) 100 g (PLA): 20 g (AgNO_3_): 10 g (PVP), hereafter denoted as PLA/Ag/PVP (rec-03) powder and the filament produced with this powder, (**f**) 100 g (PLA): 10 g (AgNO_3_): 5 g (PVP), hereafter denoted as PLA/Ag/PVP (rec-04) powder and the filament produced with this powder.

All powder mixtures prepared for the four different recipes appearing in white color were fed into a Noztek Pro (Shoreham, UK) single screw extruder preheated at 230 °C and processed at the same temperature for 5 min prior to being extruded to 3D printing filaments (Figure 1c). It is worth mentioning that a direct proof for the successful reactive melt mixing process and the in-situ generation of AgNPs in the PLA matrix was the observed change in color of all extruded filaments, namely, from white colour of the powder mixtures fed into the extruder to typical silver colour, characteristic colour of silver “in-bulk” and/or AgNPs at a high concentration. The same procedure was followed for all PLA/Ag/PEG and PLA/Ag/PVP extruded filaments (filaments were dried at 50 °C for four hours) (Figure 1d), while the process flow for the different recipes is schematically shown in Figure 1. Samples from the PLA/AgNP nanocomposites filaments’ resulting from the four different recipes followed were initially analyzed (diameter, discontinuities, etc.) before being used further for 3D printing (Figure 2). Regarding the measured filaments diameter, the average value was 1.74 mm and the standard deviation was ±0.04 mm.

### 2.3. 3D Printing of PLA/AgNP Nanocomposite Filaments

The commercially available desktop 3D printer Intamsys Funmat HT was used in all cases. All specimens were 3D printed in the horizontal orientation and American Society for Testing and Materials (ASTM) D638-02a standard (type V specimens with 3.2 mm thickness) dog-bone shaped specimens were manufactured. As the standard requires, five specimens were produced for neat PLA and for each one of the four different recipes.

The specimens were manufactured with the following 3D printing parameters: 100% solid infill, 45 degrees deposition orientation angle, 0.2 mm layer height, and 230 °C 3D printing nozzle temperature.

### 2.4. Characterisation Techniques

Raman spectroscopy was performed with a Labram HR-Horiba (Horiba Scientific, Kyoto, Japan) scientific micro-Raman system. All spectra were acquired in the back-scattering geometry with a 514.5 nm line of an Ar^+^ ion laser operating at 1.5 mW power at the focal plane. In order to facilitate the excitation light onto the sample’s surface as well as collecting the back-scattering Raman activity, a 50× long working distance objective has been utilized as part of an optical microscope set-up.

Thermogravimetric analysis (TGA) studies were carried out using a NETZSCH STA 409C/CD (NETZSCH Gerätebau GmbH, Selb, Germany). The experiments have been conducted from ambient (25 °C) up to 800 °C in oxygen atmosphere with a heating rate of 10 K/min, while for the temperature calibration, Curie point standards were utilized.

The sessile drop method was employed to investigate the wettability of 3D printed PLA/AgNP with different layer thickness (100 and 300 µm layer thickness) by H_2_O using the Dataphysics OCA 20 (Dataphysics, Filderstadt, Germany) contact angle analyser system. The environment experimental conditions for all measurements were 65% relative humidity at 25 ± 1 °C. All samples were kept for drying at 60 °C under vacuum overnight before performing the contact angle measurements. A droplet of deionized water with 2 μL volume was dispensed onto the surface of the 3D printed samples, while the droplet profile was recorded with a CCD video camera after 5 s in all measurements. In order to form the water droplets onto the 3D printed samples (with variable printed layer thickness), from which the respective contact angles have been determined using each single droplet profile, a microsyringe was employed. The reported values are representative of at least five different samples of which three measurements were performed at different positions, as a means to acquire statistically valid values.

Optical microscopy (OM) has been performed with Keyence VHX-6000 optical microscope (Keyence, Itasca, IL, USA). Scanning electron microscopy (SEM) investigations were performed using a FEI NanoSem 200 (FEI, Eindhoven, The Netherlands) at an accelerating voltage of 2 kV. Prior to the SEM analysis, a thin layer (3 nm) of platinum was deposited by sputtering to avoid charging effects as reported elsewhere [51,52].

### 2.5. Antibacterial Activity

The antibacterial activity of PLA/AgNP 3D printed samples was tested against Gram-positive Staphylococcus aureus (*S. aureus*, ATCC 25923), as well as Gram-negative Escherichia coli (*E. coli*, ATCC 25922) bacteria strains, according to the standard shake flask method (ASTM-E2149-01). The antibacterial tests have been carried out in a typical microbiology laboratory. All glassware, materials and relevant cell culture hardware have been sterilized before the experiments using an autoclave at 121 °C/1.5 atm for 20 min. Moreover, all cultures have been grown in petri dishes in a laminar flow hood (LFH). The laboratory temperature maintained between (23–25 °C) using an air conditioning facility for the microbiology laboratory. The growth of bacteria in liquid cultures was determined by measuring the optical density at 600 nm (OD600) with a Helios Epsilon Photometer (Thermo Scientific, Waltham, MA, USA).

This method is known to provide quantitative data for measuring the reduction rate in a specific CFU number at a specific time. The CFU has been expressed further into average colony forming units per millilitre (CFU·mL^−1^) of buffer solution in the flask. Specifically, *S. aureus* and *E. coli* cultures of bacteria were grown on nutrient agar overnight, while they have been transferred then into a nutrient broth (NB) with an initial optical density (OD) of 0.1 at 660 nm and allowed to grow at 37 °C and 110 rpm. Upon reaching an OD of 0.3 at 660 nm which is known as the beginning of the logarithmic phase, the cultures were centrifuged and washed twice with saline at pH 6.5 to yield a final bacterial concentration of approximately 10^8^ CFU·mL^−1^. Afterwards, a PLA/AgNP small piece (~0.5 gr) and 4.5 mL of a saline solution were inserted into a vial with an inner diameter of 2.5 cm, while 500 μL of the strain cells were poured into the vial using a micropipette. In these conditions, it has been achieved an initial bacterial concentration in the vial of appr. 10^7^ CFU·mL^−1^. The bacterial suspensions were incubated and shaken then at 37 °C and 230 rpm for up to 120 min (2 h). As a final step, samples of 100 μL each were taken at a specified time (30, 60, and 120 min), diluted tenfold in saline and then transferred onto nutrient agar plates. The plates were allowed to grow then at 37 °C for 24 h to determine the number of surviving bacteria. The antimicrobial activity is reported in terms of percentage of bacteria reduction calculated as the ratio between the number of surviving bacteria before and after the contact with the control (PLA) and PLA/AgNP 3D printed samples, making use of the following formula:

(1) Bacteria reduction (%)=((A−B)/A)×100

where *A* and *B* are the average number of bacteria before and after the contact with the PLA/AgNP samples. For each bacteria strain, the experimental protocol was conducted three times (*n* = 3), while the antibacterial activity against *S. aureus* and *E. coli* after 30, 60, and 120 min of contact is reported as the mean ± standard deviation (SD). All the data were analysed also by one-way analysis of variance (ANOVA) and differences between the means were assessed with Neuman–Keuls’s multiple comparison tests to determine the significant variation of the PLA/AgNP bactericidal activity compared to the PLA control sample. Differences were considered significant at *p* < 0.05.

### 2.6. Tensile Tests

The tensile tests were performed according to the ASTM D638-02a standard, using an Imada MX2 (Northbrook, IL, USA) tensile test apparatus, equipped with standardized grips. The chuck of the tensile test machine was set at a 10 mm/min speed for testing. All specimens were tested for the determination of their tensile properties at room temperature (~23 °C).

### 2.7. Micro-Hardness Tests

Micro-hardness tests were performed according to the specifications of the ASTM E384-17 standard. The micro-Vickers method was applied, with 0.2 kg force scale (1.962 N) and 10 s indentation time. A typical 136° apex angle Vickers diamond pyramid was used as indenter. Experiments were carried out with an Innova Test 400-Vickers (Maastricht, The Netherlands) apparatus.

## 3. Results and Discussion

### 3.1. Raman Spectra of PLA/Ag/PEG and PLA/Ag/PVP 3D Printed Samples

**Figure 3:**
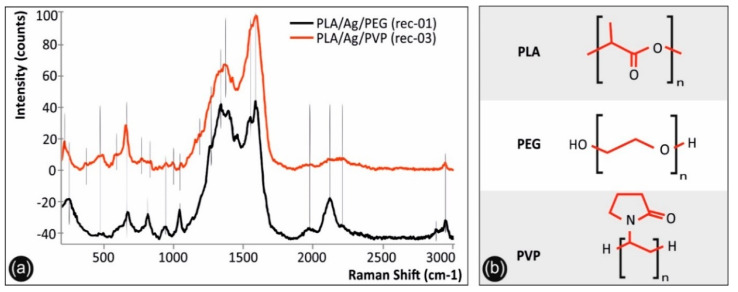
(**a**) Raman spectra of PLA/Ag/PEG and PLA/Ag/PVP 3D printed samples, and (**b**) the molecular structures of PLA, PEG, and PVP compounds of which the Raman characteristic features have been assigned.

Figure 3a shows the Raman spectra of PLA/Ag/PEG (rec-01) and PLA/Ag/PVP (rec-03) 3D printed samples, while Figure 3b the molecular architectures of PLA, PEG, and PVP. It should be mentioned that only the PLA/Ag/PEG (rec-01) and PLA/Ag/PVP (rec-03) spectra are depicted, since they have been found to exhibit identical spectral features compared to that of PLA/Ag/PEG (rec-02) and PLA/Ag/PVP (rec-04) materials, respectively. It is known from literature that silver presents a lattice vibrational mode between 50 and 300 cm^−1^, which depends on the chemical compound silver is present, i.e., as oxide, nitrate, chloride or as some other compound [55]. Specifically, the peak located at 244 cm^−1^ for the PLA/Ag/PEG spectrum, as well as a “shoulder” in the case of PLA/Ag/PVP system, corresponds to the metallic AgNPs formed, being in good agreement with the Raman spectrum of 99.99% pure silver wires reported elsewhere [56].

On the other hand, the sharp band located at 222 cm^−1^ for the PLA/Ag/PVP sample, not present in PLA/Ag/PEG sample’s spectrum, is attributed to the stretching vibrations of Ag–N [57,58], due to the formation of a chemical bond between the AgNPs and the PVP nitrogen atoms [58], or Ag–O bonds [57], due to the interaction of AgNPs with the carboxylate groups (n Ag–OCO-) of PLA [59].

In both spectra, all peaks that are attributed to PLA are depicted with continuous lines, while the specific bands assigned to the chemistry of the blended reducing agent polymeric material (PEG and PVP, respectively) are illustrated with dashed lines. In specific, both spectra show the characteristic signature of PLA presenting peaks at 465, 479, and 589 cm^−1^ (C-O-C vibration), 655 cm^−1^ (C=O stretching vibration), 936 cm^−1^ (C-COO vibration), 1252 and 1320 cm^−1^ (CH deformation vibration), 1596 cm^−1^ (asymmetric C=O stretching vibrations of carboxylate groups of PLA), and 2947 cm^−1^ (CH_3_ symmetric and asymmetric stretching vibration). The PLA/Ag/PEG spectrum has additionally some bands due to PEG blended additive located at 811 cm^−1^ (C-O-C vibration), 1038 cm^−1^ (C-C stretching vibration of the PEG backbone macromolecular chains), 1375 cm^−1^ (CH_3_ deformation vibration), 1396 cm^−1^ (CH_3_ symmetric deformation vibration), 1504 cm^−1^ (CH_2_ or O-CH_2_ vibration) and 2884 cm^−1^ (CH_3_ symmetric and asymmetric stretching vibration). The PLA/Ag/PVP spectrum has additionally some bands due to PVP blended additive located at 384 cm^−1^ (C-CH_3_ stretching vibration) 770 cm^−1^ (C-N vibration), 989 cm^−1^ (C-C stretching vibration), 1062 cm^−1^(C-CH_3_ stretching vibration), 1361 cm^−1^ (CH_2_ band vibration modes of the pyrrolidone ring in PVP) and 1385 cm^−1^ (CH_3_ deformation vibration) [60].

### 3.2. Thermogravimetric Analysis of Neat PLA and PLA/Ag Nanocomposite Filaments

**Figure 4:**
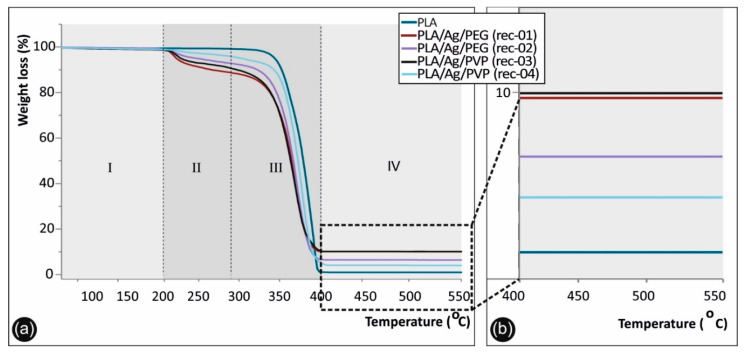
Thermogravimetric analysis (TGA) graphs of neat PLA, PLA/Ag/PEG, and PLA/Ag/PVP nanocomposite filaments fabricated under the four different recipes developed in this study in the temperature window 55 to 550 °C. (**a**) Full temperature window (55 to 550 °C) of the TGA experiment, as well as (**b**) magnified temperature window from 400 to 550 °C showing more specifically the material weight loss at 550 °C, where all the organic polymeric substances have been decomposed and the weight loss observed is attributed to the AgNPs.

Figure 4 shows the TGA graphs in the range of 55 to 550 °C for the different materials produced in this study. Namely, the neat PLA, as well as the nanocomposite PLA/Ag/PEG (rec-01 and rec-02) and PLA/Ag/PVP (rec-03 and rec-04) extruded filaments, all of which have been produced under the same experimental parameters, could be seen. Specifically, four distinct thermal decomposition windows can be observed indicated as I, II, III, and IV in the TGA figure. In the first one (I), up to ~180 °C, none of the materials exhibits any weight loss (%). Therefore, it can be deduced that all materials are stable up 180 °C.

On the contrary, from 180 °C and up to ~266 °C, the II temperature window appears, where the observed weight loss (%) is attributed to the decomposition of PEG (rec-01 and rec-02) and PVP (rec-03 and rec-04), respectively. It can be observed that PLA/Ag/PEG (rec-01) shows the same decomposition trend with the PLA/Ag/PVP (rec-03), while PLA/Ag/PEG (rec-02) similar to the PLA/Ag/PVP (rec-04). This is more precisely explained by the fact that in both cases an equal amount of PEG and PVP, respectively, was added as the macromolecular reactive melt mixing reducing agent for the creation of AgNPs in the PLA matrix. From approximately 266 °C and up to 374 °C, corresponding to the temperature window III, the onset of PLA decomposition could be observed starting from 266 °C, while at 374 °C all polymer based substance in the different materials/filament formulations has been fully decomposed (both PLA and PEG or PVP in the different recipes).

The final temperature window (IV) of 374 to 550 °C is mainly included to demonstrate the remaining material after all polymer has been decomposed, which is attributed to the generated AgNPs within the PLA matrix as solid metallic particles that are stable up to 550 °C (even up to 1000 °C that the TGA experiments left to run). The remaining material at 550 °C for the different PLA/Ag nanocomposite filament, attributed to the AgNPs grown within the PLA matrix, is 9.37% (PLA/Ag/PEG rec-01), 4.61% (PLA/Ag/PEG rec-02), 9.45% (PLA/Ag/PVP rec-03), and 4.06% (PLA/Ag/PVP rec-04), respectively. All the solid AgNP remaining material at above 400 °C for the different samples could be seen in the magnified temperature range of 360–580 °C in the right hand-side, while as mentioned above the PLA, PEG, and PVP materials have been already decomposed at lower temperatures.

### 3.3. Contact Angle–Hydrophobicity–Antiadhesive Properties-Wettability

**Figure 5:**
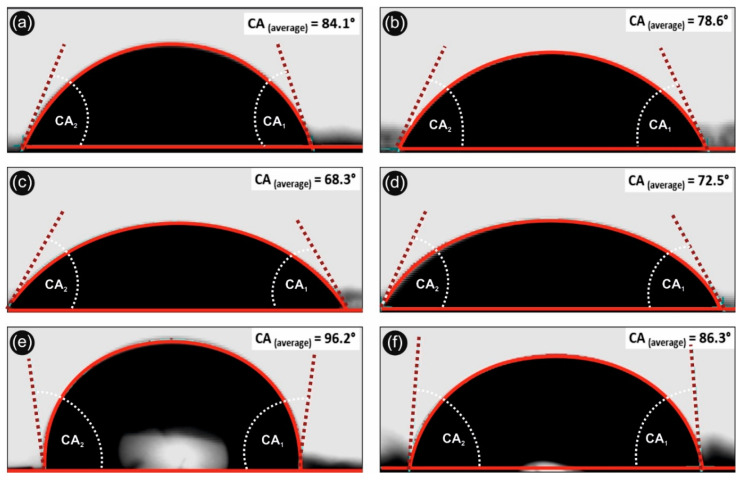
Water contact angle profiles of neat PLA, PLA/Ag/PEG (rec-01), and PLA/Ag/PVP (rec-03) 3D printed samples at 300 and 100 µm printed layer thickness, respectively, showing the hydrophobic and anti-adhesive properties of the different 3D printed objects in this work (CA_2_: contact angle 2; CA_1_: contact angle 1; CA_average_: contact angle average value in “°” from CA_1_ and CA_2_). (**a**,**b**): H_2_O contact angle of neat 3D printed PLA with 100 and 300 µm printed layer thickness, respectively. (**c**,**d**): H_2_O contact angles of PLA/Ag/PEG (rec-01) with 100 and 300 µm printed layer thickness, respectively. (**e**,**f**): H_2_O contact angle of PLA/Ag/PVP (rec-03) with 100 and 300 µm printed layer thickness, respectively.

Figure 5 shows the water contact angle onto the 3D printed different samples. Namely, Figure 5a,b correspond to the neat 3D printed PLA with 100 and 300 µm printed layer thickness, respectively. Figure 5c,d represent the H_2_O contact angles of PLA/Ag/PEG (rec-01) with 100 and 300 µm printed layer thickness, respectively. Finally, Figure 5e,f show the H_2_O contact angle of PLA/Ag/PVP (rec-03) with 100 and 300 µm printed layer thickness, respectively.

It can be seen that in the case of PLA and PLA/Ag/PVP 3D printed samples, the contact angle increases with increasing the 3D printed object micro-roughness, achieved by decreasing the printed object layer thickness from 300 to 100 µm. This is more precisely explained by the well-known Cassie–Baxter model that drives the wetting phenomena of surfaces by different liquids taking into account different ranges of surface roughness i.e., micro-roughness, nano-roughness, etc. [61]. Moreover, by decreasing the roughness of surfaces, i.e., creating nanostructured surfaces with specific features, one can achieve superhydrophobic surfaces following biomimetic nanostructured surfaces as for instance this of the well-known lotus leaf (otherwise known as “lotus leaf effect” to achieve super-hydrophobicity, anti-adhesive and self-cleaning surfaces). On the other hand, the H_2_O contact angle of PLA/Ag/PEG (rec-01) decreases for the 100 µm printed layer thickness sample, due to the existence of PEG blended material within the PLA matrix that is hydrophilic water soluble material. Interestingly enough, all 3D printed samples exhibit a hydrophobic behavior, namely PLA, PLA/Ag/PEG, and PLA/Ag/PVP which is important for their use in biomedical applications with anti-adhesive properties, preventing from the microorganism attachment and colonization. Specifically, the PLA/Ag/PVP 3D printed specimen with the smallest printed layer thickness dimension (100 µm), exhibited the highest H_2_O contact angle (>100°) which is important both as antiadhesive surface as well as easily to be cleaned since bacteria and biofilm cannot be created due to the required hydrophilic surface.

### 3.4. Optical Microscopy Investigations

**Figure 6:**
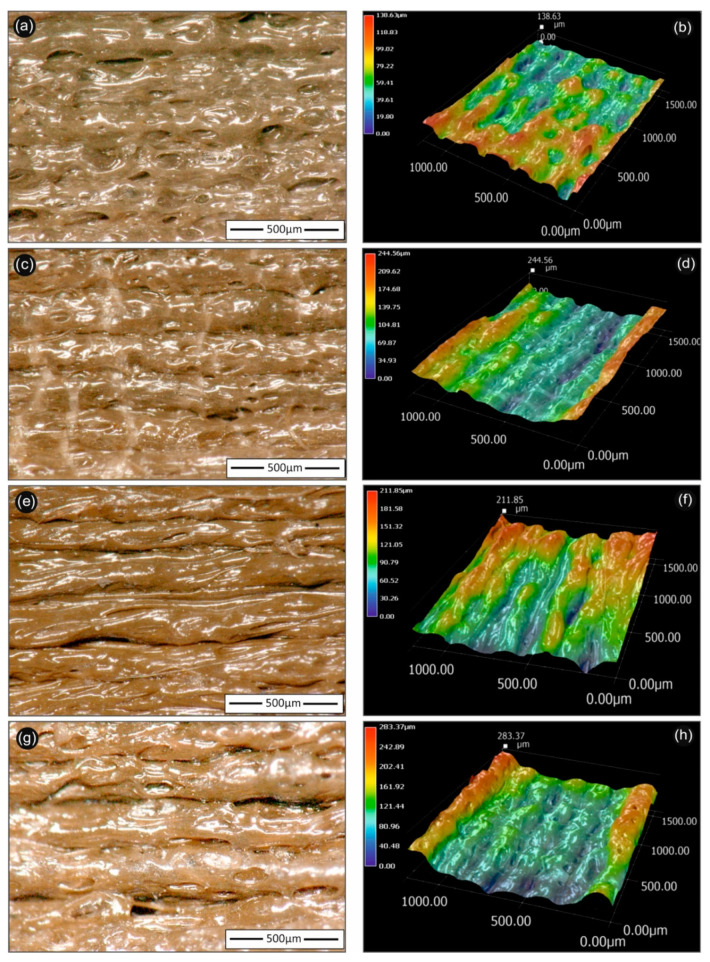
Optical microscopy 2D (left hand-side) and 3D (right hand-side) images of 3D printed PLA/AgNP nanocomposites (at 100 µm printed layer thickness), utilizing PLA/Ag/PEG (rec-01)—(**a**,**b**); PLA/Ag/PEG (rec-02)—(**c**,**d**); (PLA/Ag/PVP (rec-03)—(**e**,**f**); and PLA/Ag/PVP (rec-04)—(**g**,**h**) extruded nanocomposite filaments, respectively. The 500 µm scale bar is inserted and shown in each figure in the left hand-side images, while the respective 3D images are shown in the right hand-side with the corresponding color scale bar indicating the micro-roughness of each sample.

Figure 6 summarizes the optical microscopy images of the 3D printed PLA/AgNP nanocomposite samples using PEG (recipe 01 and 02), as well as PVP (recipe 03 and 04) as reactive melt blending additive for the reduction of Ag^+^ to metallic Ag^0^ (AgNPs). All images were acquired from 3D printed samples fabricated with 100 µm printed layer thickness. It can be observed that all 3D printed samples exhibited micro-roughness ranging from few microns to R_max_ of approximately 300 µm. Moreover, all samples showed homogeneous printed layer thickness with very few voids and discontinuities in the interlayers attributed both to the optimum printing parameters used in our study, as well as the high quality of the produced PLA/AgNP nanocomposite filaments, i.e., structural homogeneity and diameter uniformity.

### 3.5. Microstructure Investigation via SEM Analysis

**Figure 7:**
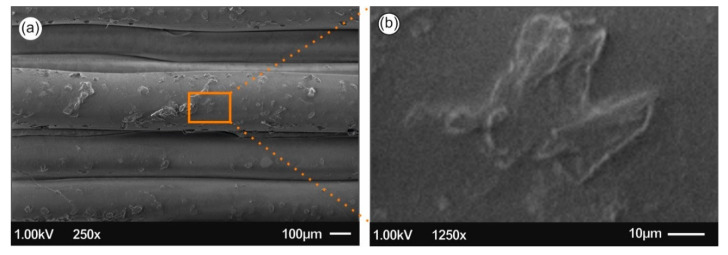
Scanning electron microscopy (SEM) images of the side-surface microstructure and morphology for the different 3D printed samples in this study at two different magnifications (all 3D printed samples with 300 µm printed layer thickness): Neat PLA SEM image at 250× magnification (**a**) and 1250× magnification (**b**), respectively.

**Figure 8:**
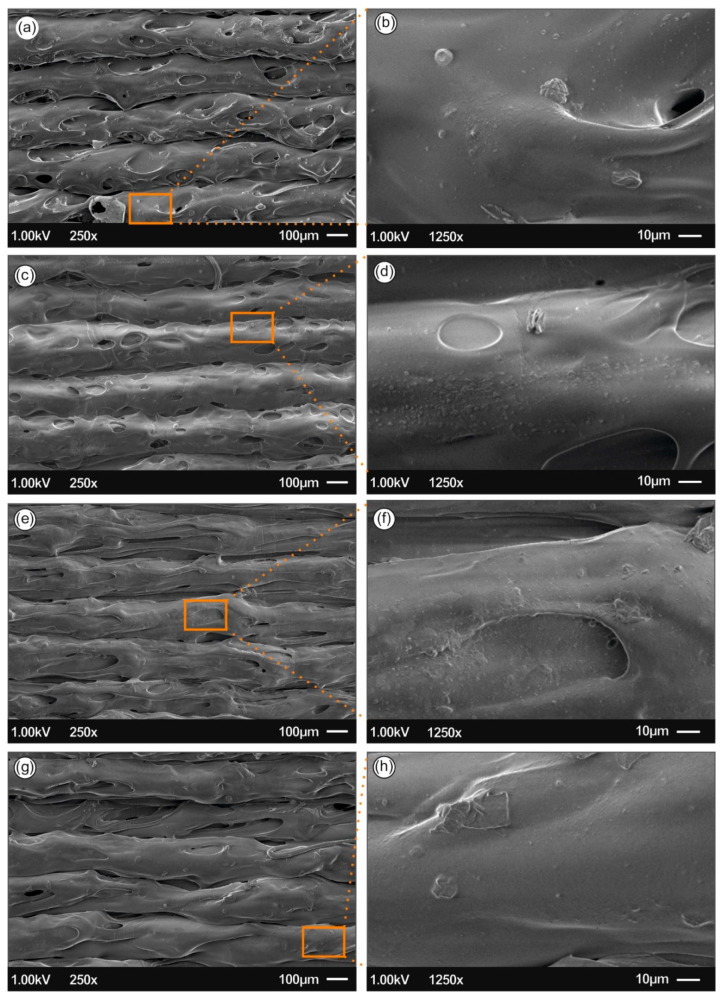
SEM images of the side-surface microstructure and morphology for the different 3D printed samples in this study at two different magnifications (all 3D printed samples with 300 µm printed layer thickness): (**a**,**b**) PLA/Ag/PEG using rec-01 filament, (**c**,**d**) PLA/Ag/PEG using rec-02 filament; (**e**,**f**) PLA/Ag/PVP using rec-03 filament; and (**g**,**h**) PLA/Ag/PVP using rec-04 filament.

Figure 7 and Figure 8 present the 3D printed samples microstructure investigated from the 3D printed side-surface (all samples shown with 300 µm printed layer thickness), at low magnification demonstrating the printing process features, as well as at high magnification showing the existence of AgNPs. Specifically, Figure 7a presents the microstructure of neat PLA with a layer thickness of approximately 300 μm, being in a good agreement with the resolution of the Intamsys Funmat HT 3D printer manufacturer’s technical specifications in terms of printer’s resolution. Figure 7b shows an amorphous PLA surface morphology, typical for polymeric materials.

Figure 8a,b show the 3D printed PLA/Ag/PEG nanocomposite surface morphology using filament of recipe 01, while Figure 8c,d PLA/Ag/PEG nanocomposite filament of recipe 02, where PEG has been utilized as the reactive melt blending additive for the reduction of Ag^+^ to metallic Ag0 (AgNPs). On the other hand, Figure 8e,f shows the 3D printed PLA/Ag/PVP nanocomposite surface morphology using filament of recipe 03, while Figure 8g,h shows the PLA/Ag/PVP nanocomposite filament of recipe 04, where PVP was employed as the reducing agent.

For all samples, it can be observed a homogeneous 3D printed layer thickness as well as high quality of bonding of the layers, indicating: (i) the optimum set of the selected printing parameters, as well as (ii) the high quality of the produced PLA/AgNP nanocomposite filaments, i.e., structural homogeneity and diameter uniformity produced in our study by reactive melt-blending process.

Moreover, from the SEM images it can be further deduced that high quality 3D printed objects have been manufactured with good adhesion between the layers, which could most likely result further in high mechanical performance 3D printed components. Finally, at high magnification images for all cases of the 3D printed PLA/AgNP nanocomposites, the existence of AgNPs with sizes ranging from 50–100 nm blended and homogeneously distributed within the PLA matrix can be observed.

### 3.6. Bactericidal Tests

The antibacterial activity of the PLA/AgNP 3D printed nanocomposites with 100 µm printed layer thickness, utilized for the FFF 3D printing process filaments produced by recipe-01, recipe-02, recipe-03, and recipe-04, was examined by reduction in *S. aureus* and *E. coli* bacteria viability (%) at 30, 60, and 120 min duration of contact. It should be mentioned that differences were considered significant at *p* < 0.05. It can be seen that *E. coli* cultures of the bacteria were eradicated completely after 120 min of treatment (*p* < 0.05) with PLA/Ag/PEG (rec-01) and PLA/Ag/PVP (rec-03), while being eradicated (>90%) using PLA/Ag/PEG (rec-02) and reaching >70% in the reduction of bacteria viability by PLA/Ag/PVP (rec-04).

The observed required time window of 120 min to reduce the initial *S. aureus* and *E. coli* amount of CFU to zero is governed by the velocity of Ag^+^ ions release, which are responsible for the antibacterial activity (*p* < 0.05). It is worth mentioning that even in 60 min of *S. aureus* and *E. coli* treatment with PLA/Ag/PEG (rec-01) and PLA/Ag/PVP (rec-03), more than 80% of the bacteria have been eradicated. At the same time, PLA/Ag/PEG (rec-02) and PLA/Ag/PVP (rec-04) consisting 3D printed objects, exhibited remarkably less antibacterial activity due to possibly less amount of AgNPs existent onto the outer surface of the PLA/AgNP 3D printed objects, being in good agreement with the TGA experiments showing the generated AgNP solid content as well as the utilized AgNO_3_ precursor used in the reactive melt-mixing recipe.

Overall, the 3D printed manufactured PLA/AgNP samples exhibit excellent antibacterial properties with the greatest bactericidal effect on *E. coli* (>98%) for both the PLA/Ag/PEG (rec-01) and PLA/Ag/PVP (rec-03), and the lowest on *S. aureus* with 74.9% for the PLA/Ag/PEG (rec-01) and 68.9% for the PLA/Ag/PVP (rec-03), respectively, at 120 min of contact (*p* < 0.05). It is worth mentioning that the PLA/Ag/PEG showed higher antibacterial performance both against *E. coli* as well as *S. aureus* at the different durations of contact. This could be more precisely explained by the fact that the PEG reducing agent additive in the system is more hydrophilic (shown also by the water contact angle experiments) so that the bacteria strains could be easier attached onto the sample surface to start further their colonization, while at the same time the Ag^+^ release is more pronounced in the PLA/PEG blended system killing the bacteria more effectively.

**Figure 9:**
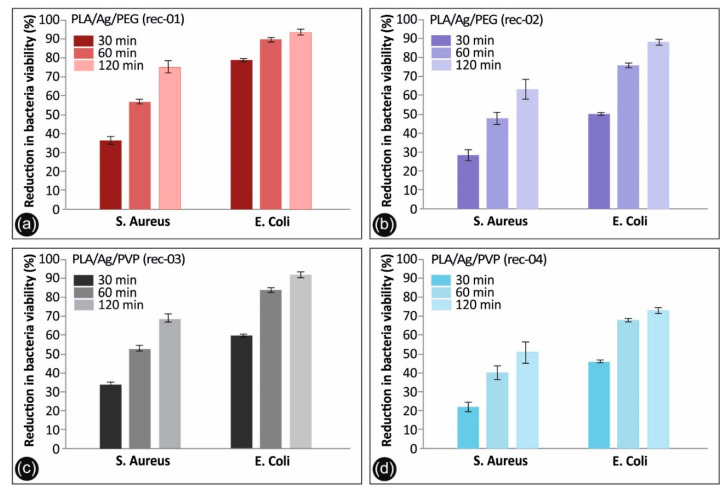
Antibacterial activity of (**a**) PLA/Ag/PEG (rec-01), (**b**) PLA/Ag/PEG (rec-02), (**c**) PLA/Ag/PVP (rec-03) and (**d**) PLA/Ag/PVP (rec-04) 3D printed PLA/AgNP nanocomposites with 100 µm 3D printed layer thickness, against *S. aureus* and *E. coli* after 30, 60, and 120 min of contact. Mean values do not differ significantly (*p* < 0.05).

**Figure 10:**
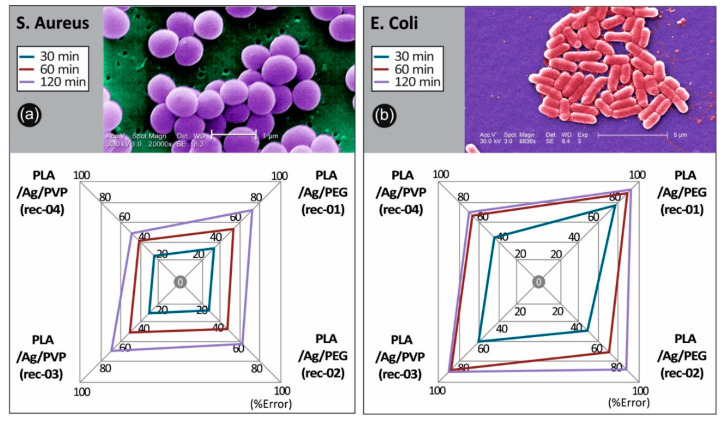
Comparative spider graphs for the antibacterial behavior for the four 3D printed PLA/AgNP nanocomposites produced in this work (with 100 µm 3D printed layer thickness) against (**a**) *S. aureus* and (**b**) *E. coli* after 30, 60, and 120 min of contact. Values are mean. Mean values do not differ significantly (*p* < 0.05).

The results obtained in our study regarding the differences in bacteria viability between Gram-positive and Gram-negative strains using AgNPs as the antimicrobial agent, are in good agreement with findings recently reported by Huq et al. [62]. Specifically, in this work the growth curves of Gram-positive *S. aureus* Gram-negative *E. coli* bacteria strains cultured in R2A broth with various concentrations of AgNPs have been reported, showing a higher AgNP susceptibility of Gram-negative bacteria compared to that of Gram-positive bacteria. This was further explained due to distinctions in the composition of the bacteria cell wall, which was similarly reported also in another study [63]. PLA surfaces of bare PLA retractor exhibited no bactericidal effect. The detailed results are presented in Table 1 (values shown represent the means ± SD of triplicate measurements; *n* = 3) and Figure 9 and Figure 10.

### 3.7. Tensile Properties and Fracture Surface Analysis

Tensile test experiments were performed on 3D printed dog bone-shaped samples manufactured from neat PLA extruded filament, as well as nanocomposite PLA/AgNP filament from the PLA/Ag/PEG (rec-01), PLA/Ag/PEG (rec-02), PLA/Ag/PVP (rec-03), and PLA/Ag/PVP (rec-04).

**Figure 11:**
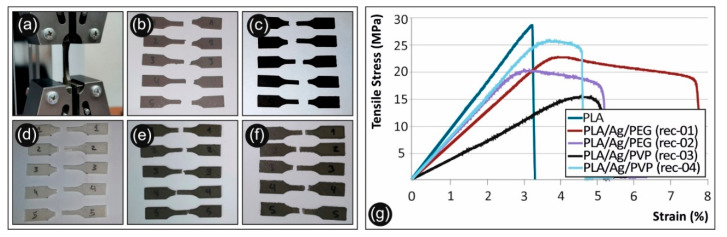
(**a**) Tensile test of a specimen, (**b**) Failed PLA/Ag/PEG (rec 1) specimens after the tensile tests, (**c**) Failed PLA/Ag/PEG (rec 2) specimens after the tensile tests, (**d**) Failed pure PLA specimens after the tensile tests, (**e**) Failed PLA/Ag/PVP (rec 3) specimens after the tensile tests, (**f**) Failed PLA/Ag/PVP (rec 4) specimens after the tensile tests, (**g**) Tensile stress vs. strain graphs for the neat PLA and the four recipes prepared in this work. All graphs are from the no 1 specimen of each case studied.

**Figure 12:**
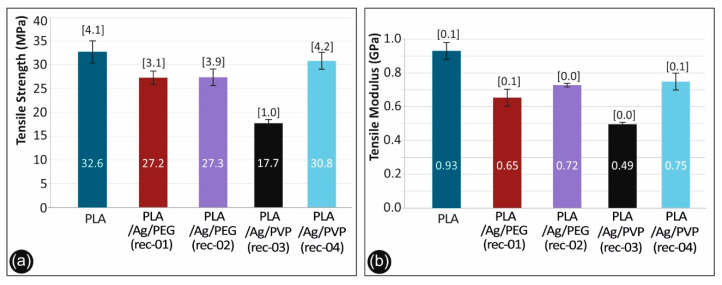
(**a**) Comparative tensile strength graph and (**b**) tensile mod. of elasticity for all the materials studied.

Figure 11 shows typical stress–strain curves derived and calculated from the tensile testing of the nanocomposites. Both tensile strength and moduli results with the corresponding standard deviations are plotted in Figure 12a,b respectively for the different sample formulations.

As it can be observed, there is a knock-down effect on the tensile strength of the 3D printed PLA/Ag/PEG (rec-01), PLA/Ag/PEG (rec-02), PLA/Ag/PVP (rec-03), and PLA/Ag/PVP (rec-04) as compared to the neat PLA 3D printed specimens. This is more precisely attributed to the low molecular weight PEG (Mn = 4600 g/mol) and PVP (Mn = 10,000 g/mol) additives in PLA/Ag/PEG (rec-01 and rec-02) as well as PLA/Ag/PVP (rec-03 and rec-04), respectively; utilized as the macromolecular reducing agents for the creation of AgNPs in the four different blend recipes. Moreover, it can be seen also that the effect of strength reduction is more prominent for the PLA/Ag/PEG (rec-03) as compared to the reference PLA. In general, the tensile strength knock-down is not significant as compared to the neat PLA reference material for all recipes tested, except the PLA/Ag/PVP (rec-03). Due to the reasons explained above, a minor decrease in the mechanical properties was expected. The main aim of this work was not to increase the mechanical properties of the produced nanocomposites, but to produce nanocomposites with improved antibacterial and other properties and test the mechanical behavior of the parts manufactured with these nanocomposites, which were expected to be similar to the polymer matrix material. This is achieved with the recipes implemented and tested in this work.

The generation of AgNPs by reactive melt mixing extrusion resulted in a slight decrease of the modulus of elasticity for all the 3D printed PLA/Ag/PEG (rec-01), PLA/Ag/PEG (rec-02), PLA/Ag/PVP (rec-03) and PLA/Ag/PVP (rec-04) specimens when compared to the neat PLA 3D printed sample.

**Figure 13:**
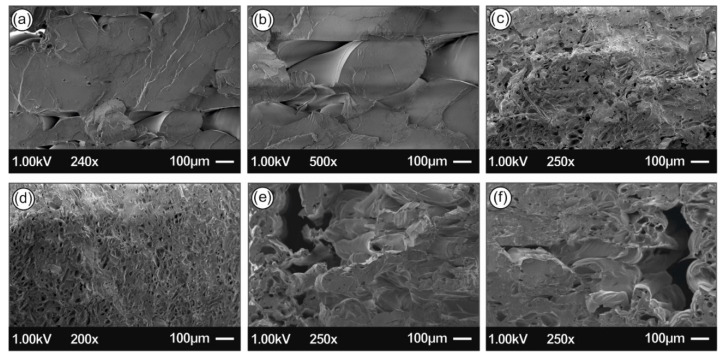
SEM images of the tensile test fractured areas (**a**,**b**) Neat PLA, (**c**) PLA/Ag/PEG rec-01 specimen, (**d**) PLA/Ag/PEG rec-02 specimen, (**e**) PLA/Ag/PVP rec-03 specimen, and (**f**) PLA/Ag/PVP rec-04 specimen.

Figure 13 shows SEM images of the tensile test fractured areas of the neat PLA specimens and the specimens manufactured with the four different recipes of this study. Images show a more brittle behavior of the neat PLA, when compared to the four nanocomposites, which is in agreement with the stress- strain graphs produced during the experiments of this work. The more ductile behavior of the recipe 1 nanocomposite is also verified by the images taken in the fracture areas of the specimens. Also, it was evident that extrusion produced nanocomposites with no internal structuring faults, since a uniform structure can be observed in all different materials developed in this work.

### 3.8. Micro-Hardness Properties of 3D Printed PLA and PLA/AgNP Nanocomposites

**Figure 14:**
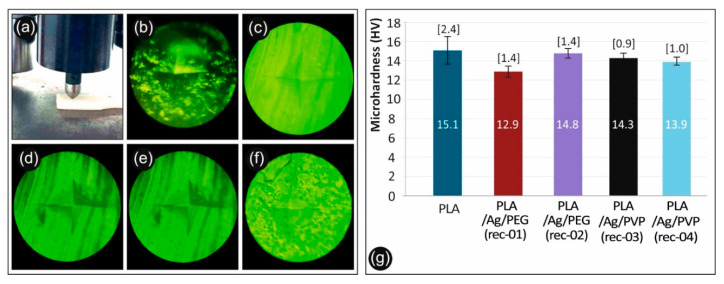
(**a**) Measuring the Vickers Micro-Hardness of a specimen, (**b**) PLA/Ag/PEG (rec 1) specimen, (**c**) PLA/Ag/PEG (rec 2) specimen, (**d**) Pure PLA specimen, (**e**) PLA/Ag/PVP (rec 3) specimen, (**f**) PLA/Ag/PVP (rec 4) specimen, and (**g**) Micro-Hardness Vickers results of neat PLA and the four recipes prepared in this work.

The calculated microhardness of the 3D printed manufactured samples consisting of neat PLA extruded filament, as well as nanocomposite PLA/AgNP filament from the PLA/Ag/PEG (rec-01), PLA/Ag/PEG (rec-02), PLA/Ag/PVP (rec-03), and PLA/Ag/PVP (rec-04) are depicted in Figure 14g. As it can be seen, the microhardness of the neat PLA is marginally higher than the microhardness of the four nanocomposites, showing that these specific fillers effect on the microhardness of the PLA polymer matrix is negligible. Some microhardness variation and decrease for the PLA/AgNP nanocomposite samples could be attributed to some plasticization effect that has been induced to the PLA due to the blended macromolecular chains of the PEG (rec-01 and rec-02) and PVP (rec-03 and rec-04) that have been utilized as reducing agents in the melt state for the in-situ growth of the AgNPs.

## 4. Conclusions

Since PLA has been proven to be safe for biomedical applications, cost effective, safe, and an environmentally suitable material for printing, 3D printed antimicrobial and anti-adhesive objects have been produced herein via the 3D printing FFF 3D printing process. PLA/AgNP antimicrobial filaments have been produced by reactive melt mixing, via blending of (i) PLA, AgNO_3_, and PEG, as well as (ii) PLA, AgNO_3_, and PVP. PEG and PVP have been utilized as alternative macromolecular reducing agent polymeric materials in both cases. The PLA as the polymer matrix and AgNO_3_ as the AgNP precursor salt. AgNPs have been generated in the PLA polymer matrix, yielding extremely efficient antimicrobial (AM) objects as determined by the reduction in Staphylococcus aureus (*S. aureus*) and Escherichia coli (*E. coli*) bacteria viability (%) after being exposed for 30, 60, and 120 min, respectively. Such behavior is in the direction of mimicking nature mechanism to kill these specific types of bacteria, while the same effect is possible to be expected in other types of bacteria, too, and can expand the current study in the future in this direction.

The advantages of the current study’s protocol lie in the fact that: (i) molecularly the Ag^+^ stemming from the AgNO_3_ dissociated in the polymer melt are distributed in volume of the PLA melt getting reduced further in-situ during the melt-mixing compounding process, (ii) it is a clean process avoiding mixing of e.g., Ag nanopowders, (iii) ready-made AgNPs are known to be expensive, (iv) AgNPs could agglomerate during processing and affect the rheology of the system increasing the melt viscosity, and (v) there is no guarantee or full characterization about the NP quality (size, monodispersity), while in many cases of commercially available AgNPs the suppliers do not disclose the particle surface chemistry, affecting both the nanodispersion process as well as the nanocomposites’ antimicrobial properties. It could be envisaged that the proposed 3D printed AM objects demonstrated in this work with tunable anti-adhesive properties varying the 3D printed object printed layer thickness, could possibly substitute a great number of metallic objects, i.e., titanium consisting nosocomial equipment, etc. with a much lower cost.

## Figures and Tables

**Table 1:** Antibacterial activity of 3D printed PLA/AgNP nanocomposites (@100 µm printed layer thickness) against *S. aureus* and *E. coli* after Different Incubation Times (*n* = 3, *p* < 0.05).

Reduction in Viability (%)				
Sample Formulation		30 min	60 min	120 min
PLA/Ag/PEG (rec-01)	*S. aureus*	36.42 ± 2.02	57.20 ± 0.88	74.91 ± 3.78
	*E. coli*	79.17 ± 0.55	90.01 ± 0.95	94.86 ± 1.22
PLA/Ag/PEG (rec-02)	*S. aureus*	28.23 ± 3.08	47.45 ± 3.88	62.96 ± 5.78
	*E. coli*	49.17 ± 0.52	76.68 ± 0.95	88.97 ± 1.45
PLA/Ag/PVP (rec-03)	*S. aureus*	33.42 ± 3.02	52.20 ± 0.81	68.93 ± 3.32
	*E. coli*	60.17 ± 0.85	83.85 ± 0.95	92.97 ± 1.12
PLA/Ag/PVP (rec-04)	*S. aureus*	22.35 ± 2.68	40.21 ± 3.88	50.95 ± 5.78
	*E. coli*	46.17 ± 0.88	68.08 ± 0.99	73.28 ± 1.95
